# An efficient method for link prediction in weighted multiplex networks

**DOI:** 10.1186/s40649-016-0034-y

**Published:** 2016-11-05

**Authors:** Shikhar Sharma, Anurag Singh

**Affiliations:** 1 Cluster Innovation Centre, University of Delhi, Delhi, 110007 India; 2 Department of Computer Science and Engineering, National Institute of Technology Delhi, Delhi, 110040 India

**Keywords:** Link prediction, Multiplex networks, Complex networks, Weighted networks

## Abstract

**Background:**

A great variety of artificial and natural systems can be abstracted into a set of entities interacting with each other. Such abstractions can very well represent the underlying dynamics of the system when modeled as the network of vertices coupled by edges. Prediction of dynamics in these structures based on topological attribute or dependency relations is an important task. Link Prediction in such complex networks is regarded useful in almost all types of networks as it can be used to extract missing information, identify spurious interactions, and evaluate network evolving mechanisms. Various similarity and likelihood-based indices have been employed to infer different topological and relation-based information to form a link prediction algorithm. These algorithms, however, are too specific to the domain and do not encapsulate the generic nature of the real-world information. In most natural and engineered systems, the entities are linked with multiple types of associations and relations which play a factor in the dynamics of the network. It forms multiple subsystems or multiple layers of networked information. These networks are regarded as Multiplex Networks.

**Methods:**

This work presents an approach for link prediction in Multiplex networks where the associations are learned from the multiple layers of networks for link prediction purposes. Most of the real-world networks are represented as weighted networks. Weight prediction coupled with Link Prediction can be of great use. Link scores are received using various similarity measures and used to predict weights. This work further proposes and testifies a strategy for weight prediction.

**Results and Conclusions:**

This work successfully proposes an algorithm for Weight Prediction using Link similarity measures on multiplex networks. The predicted weights show very less deviation from their actual weights. In comparison to other indices, the proposed method has a far low error rate and outperforms them concerning the metric performance NRMSE.

## Background

Many systems and their interactions can be modeled as an abstraction which provides a suitably accurate representation of their dynamics. These dynamics can be simulated to discover new properties, interactions, and different representations. They can also be used to forecast or predict behavior. These entities can be modeled as nodes in a graph where the links/edges are representative of the interactions/relationships between the individuals. For instance, a simple friendship network can be represented with the nodes representing the individual under study and the edges representing if two individuals are friends. Alternatively, a normalized weight can be added to each link to denote the amount of friendship between two individuals derived from some other factors. Link prediction or forecasting the formation of a link in such systems with their abstraction as complex networks is useful in multiple ways. Link prediction attempts to formulate a likelihood of the existence of a link between two nodes. In many networks, whether a link exists or may exist in future is a matter of time and resource consuming experiments. Link prediction is an adequate substitute in such cases as the focus can be shifted to the links according to a higher likelihood obtained from a sophisticated model. In the age of growing network and demand for efficient network analysis mechanisms, link prediction becomes necessary in addressing the problem of missing link. Moreover, link prediction forms the basis of various recommended systems which are used in online marketing, e-commerce services, and many social networks. Link prediction in terrorist communication networks can help predict and intercept vital information about the issue of national security. Furthermore, link prediction also comes into play in various situations such as to strategize an efficient transport network and to study genetically transferred diseases. These networks can be studied and may contain many different aspects. They may be represented in the form of temporal networks [1] or information can also be visualized in the form of multilayer networks [2].

Various strategies have been adopted for link prediction. Some of the strategies are based on the local topological information from the network corresponding to an index of evaluation. Common neighbor (CN) [3] algorithm is one such strategy which relies on the fact that more the number of common neighbors of two unconnected nodes have at a given time, higher is the likelihood of them forming a link in future. Salton index [4] normalizes the common neighbor index by the degree of individual nodes. A new strategy has been proposed for predicting the missing links which also takes into account the number of links between two sets of uncommon neighbors of given nodes, in addition to their common neighbors [5]. Other local similarity-based indices like Jaccard index [6], preferential attachment index [7], hub depressed index, hub promoted index, [8] have also been used for link prediction. Indices are taking in account the global information from graphs (path ensemble and average commute time) such as Katz index [9] and cosine-based methods have also been successfully employed for link prediction. Strategies using the attributes of a node have been proposed to a good effect [10]. Furthermore, Bliss et al. [11] have successfully employed evolutionary algorithm to combine various indices to get an optimized result, whereas He et al. [12] have used the OWA operator for the purpose of combination. A brief description of some of these methods can be found in the next section. However, most real-world networks are better visualized in the form of multiple types of relationships rather than on a single one. For Instance, consider prediction of a social network attachment between two entities. The prediction would be more likely caused by a combination of factors such as mutual interests, spatial presence at certain times, common acquaintances. The target to be predicted is not just based on a single parameter but consists of a composite informational paradigm. For Instance, two persons being friends depend not just only on whether they share the same interests but also on other aspects such as time spent together, common acquaintances, and various other factors. It is not realistic to just establish and determine relationships based on a single parameter. A multiplex network is a network in which every pair of nodes can be connected by is a pair of nodes with different types of edges and can be visualized in the form of multiple layers. Such networks contain various types of information but are sparse in nature. A multiplex network stores each type of information as a separate layer and thus encapsulates a significant number of different relationships between the same entities. The current algorithms are highly network and context specific. They tend to rely on some underlying assumptions of the network rather than inferring those from the network itself. Their performance also relies on a large number of edges. Thus, these algorithms give mixed results on multiplex networks and are unable to use the information from different layers in an efficient manner. In this work, we propose an approach that can be utilized to predict links in multiplex networks where the extension of pre-existing algorithms is not viable. Not much of work has been done in this regard, and recent works focus on a particular type of social network and not on a multiplex network in general. The importance of weights in networks is well known. The work proposes a weight prediction algorithm that depends on score assignment by the proposed likelihood algorithm to detect edge strength and similarity. These scores are used in a normalized manner to predict weights in a network. The normalization takes into account any significant differences between scores of edges under consideration. The methodology is compared to other indices based on normalized root-mean-square error as the performance metric. It is the normalized square root of the squared sum of differences in weight prediction over some test cases. This work utilizes multiple sources of information, and combines them to make an optimal prediction strategy. It is context and network independent. It operates on any network where layers exhibit a positive co-relation. The results depict the strength regarding a very little error. It is a scalable approach combining the idea of link and weight prediction and can be operated in parallel for networks with million of nodes.

The paper is organized as follows: In second section, various strategies for link prediction are discussed; in third section, a model for multiplex network is presented; in fourth section, a methodology with the help of algorithms for link prediction is proposed; in fifth section, results and analysis of the proposed algorithms are discussed; in sixth section, weights of the links are taken into consideration and a method for the weight prediction for the predicted link with the results and analysis is proposed; and in seventh section, a summary of the the analysis results of this research is provided.

## Conceptual formalization of link prediction

### Problem representation

Let a layer of network be represented by a graph *G*(*V*, *E*) where *V* is the set of nodes and *E* is the set of edges. Let |.| represent the cardinality of a set. Hence, we have |*V*| as the number of nodes and |*E*| as number of edges in the graph. Let *n* represent the number of nodes in the graph (|*V*|) and *U* represent the universal set containing all $\frac{n.(n-1)}{2}$ edges of the graph. The problem lies in assigning a likelihood to the existence of $|U|-|E|$ missing edges in the graph.

For a multiplex graph, let *L* represent the set of all layers. Between any two nodes, there can be |*L*| types of links. Some nodes may be totally disconnected in certain layers. The layer on which prediction has to be made is named as the target layer and represented by $L^{T}$. Clearly, $L=L^{T}UL^{P}$ and $L -L^{T}$ is $L ^{P}$. The problem is to assign likelihood to a link in $L^{T}$ based on information from the complete graph. It is important to note that $L^{T}$ and $L^{P}$ are used here as a set notation depicting the operations on corresponding set of vertices and edges. Similarly, the set of other layers is called as predictor layer set and is denoted by $L^{P}$.

Let $\mathrm {\Omega }$ be a subset of *E* which represents the set of edges which are used for testing the link prediction algorithm and are removed from the original graph. Let $\mathrm {\kappa }$ denote the cardinality of $\mathrm {\Omega }$ which is the number of edges in the test set.

It is important to note that the set of vertices, *V* is same for all the layers but each layer has a different subset of the set of edges *E* (all possible edges between nodes in set *V*). The target problem is missing link and weight prediction in multiplex networks.

### Evaluation metrics

The link prediction algorithm assigns a likelihood score to each pair of non-observed links (set $U-E$) and the test set $\mathrm {\Omega }$. Let *S*(*i*, *j*) denote the score for some node *i*, *j* $\mathrm {\varepsilon }$ *V*. Following are the evaluation metrics that are most commonly used to test the prediction algorithm.

#### AUC

To quantify the accuracy of the prediction algorithm, the metric of an area under the receiver-operating characteristic curve (AUC) is employed [13]. It represents the probability for a randomly chosen link from the set $\mathrm {\Omega }$ to have a greater or equal score/likelihood than a randomly chosen link from the set $U-E$. Alternatively, we can exhaustively test for each element in $\mathrm {\Omega }$. If among $\mathrm {\alpha }$-independent comparisons, there are $\mathrm {\beta }$ times that a missing link has a higher score and $\mathrm {\gamma }$ times that the scores are equal, then the AUC value is calculated as per Eq. (1)

1 $$\begin{aligned} \text{AUC}= \frac{\beta + 0.5*\gamma }{\alpha } \end{aligned}$$

The degree to which the value exceeds 0.5 indicates how much better the algorithm performs as compared to pure chance.

#### Precision

All the non-observed and test links are sorted according to the likelihood scores assigned to them. Let $\mathrm {\delta }$ be the number of shortlisted links post sorting. Let $\mathrm {\epsilon }$ be the number of relevant links, that is, one of the $\mathrm {\kappa }$ links of the set $\mathrm {\Omega }$. The precision is represented by Eq. (2).

2 $$\begin{aligned} {\text{ Precision }} = \frac{\epsilon }{\delta } \end{aligned}$$

Clearly, a higher precision is representative of a higher accuracy.

## Link prediction methodologies

This section provides an overview to some of the indices used to assign a likelihood score between a pair of candidate nodes. These indices are often context specific and so is their performance across different systems. Some of the indices are described, and other are summarized in Table 1.

### Common neighbors

According to the common neighbors (CN) [3] similarity index, given two nodes, the likelihood score of the edge joining the two nodes would be higher if they have a large number of common neighbors. This introduces a sort of triangle effect. The scores assigned are then tested against the evaluation metrics.

3 $$\begin{aligned} S(i,j) =| \Gamma (i) \cap \Gamma (j) | \end{aligned}$$

where $\mathrm {\Gamma }$ represents the neighbors set for nodes $i, j \in V$.

The neighbors of a node N are all the nodes which share an edge directly with N. In this work, neighborhood is defined for all nodes within the same layer and not for nodes in a different layer.

### Salton index

Salton index [4] divides the common neighbor index by the degree of nodes. Lesser degree nodes having more common neighbors are preferred. It is basically normalization of the graph. It is commonly used in co-authorship network.

4 $$\begin{aligned} S(i,j) =\frac{| \Gamma (i) \cap \Gamma (j) | }{\text {Deg}(i).\text {Deg}(j)} \end{aligned},$$

where Deg(.) represents the degree of the node.

### Jaccard’s coefficient

Two nodes may have a lot of common neighbors due to their high degree and not because they are strongly related. Jaccard’s coefficient [6] chooses a randomly selected feature and finds a probability of its presence in a pair of nodes normalized by its presence in the Union neighborhood of those nodes. For instance, in a co-authorship network, features are taken to be the number of common neighbors, and we take an instance of a graph where nodes represent authors and links represent a co-authorship. It is intuitive that proportion of co-authors of *i* who have also co-authored *j* is a good measure for prediction of future co-authorship.

5 $$\begin{aligned} S(i,j) =\frac{| \Gamma (i) \cap \Gamma (j) | }{| \Gamma (i) \cup \Gamma (j)|} \end{aligned}$$

### Katz index

This is a global similarity index and uses the global knowledge of network to assign the likelihood scores [9]. The idea behind the Katz measure is that, the more paths there are between two vertices and the shorter these paths are, the stronger the connection. This index is based on the ensemble of all paths, which directly sums over the collection of paths and is exponentially damped by the length to give the shorter paths more weights. Common neighbor can, in fact, be shown a particular case of the Katz index [8].

6 $$\begin{aligned} S(i,j) =\sum _{l=1}^{\infty } \zeta ^{l} * |\text {paths} _{ij} ^{<l>}| \end{aligned}$$

where $\text {paths}_{ij}^{<l>}$ is the set of all paths with length *l* connecting *i* and *j* and $\zeta$ is a damping factor. A small value of $\zeta$ leads to a value close to the measurements of CN.

### Other indices

**Table 1:** Some similarity measures for link prediction

Name of the index	Description
Adamic/Adar [14]	Counting of common features by weighting rarer features more heavily
Preferential attachment	Product of degree of nodes
Katz index	Ensemble of all paths with more weight to shorter paths
Random walk with restart [15]	Steps in which a random walker reaches from one node to another
Resource allocation [16]	Assigns scores according to resource distribution between candidate vertices with common neighbors as transmitters

Various other similar indices based on topological and probabilistic models have been proposed whose discussion is out of the scope of this work.

## Multiplex networks

Monoplex networks are a network which represents a single property and its related dynamics. There is a single layer consisting of nodes and edges. They are restricted to the representation of a single property. The majority of the real-world systems exhibit dynamics that are not just an outcome of an individual property but rather are dependent on a combination of many such properties. A pair of the node may have multiple relationships which govern the overall dynamics of a network. Most recently, there have been increasingly intense efforts to investigate networks with multiple types of connections [17]. Many real-world complex systems function through multiple layers of distinct interactions among constituents and the interplay between these interaction layers [17, 18]. It was recognized decades ago that it is crucial to study systems by constructing multiple social networks using different types of ties among the same set of individuals [19]. The different types of interactions are modeled using multiplex networks, and there exist a plethora of methods for the representation and behavior inference [2].

Assumptions about properties of the networks must be limited to as much extent as possible. Instead, properties may be obtained from the network itself. If we represent each type of relationship between a pair of nodes as a separate layer, we obtain a network with multiple layers. Following is the description of the multiplex dataset. In a multiplex network, there are set of nodes. These nodes interact with each other in many different types of relationships. Accounting for the multiplex nature of dynamics in the real world can lead to a large number of discoveries and new dimensions [18, 20]. Multiplex relationships can be visualized as an edge-colored graph where each edge represents different types of relationship [2].

**Fig. 1:**
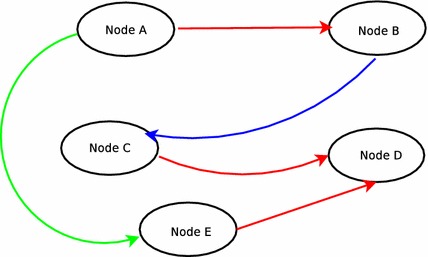
A small multiplex network with edge-colored representation

In Fig. 1, there are five nodes with three different types of relationships represented by the colors red, blue, and green. It can be considered as representative of a network of five places where each color represents whether a particular type of mode of transport [road (R), water (B), air (G)] is available to commute between these places. One may plan a journey on combinations of these factors according to different weights attributing to cost, distances, and time. Thus the optimum way of travel requires a combination of many different types of information. This information can be even more heterogeneous in nature than the above example as evident by the example dataset used for analysis in this work which models five different types of social relationships. Moreover, one can visualize such networks by preserving all nodes and keeping a particular color of edges at a certain time. This way we would have many graphs equal to the number of different colors or different types of relationships. Thereby, these can be visualized as different layers. The only inter-layer connection that is present is between the same node in both layers. Information from all such layers can be used to predict a link in another layer. The link prediction methods at present focus on considering a single type of relationship from a particular graph and ignore other factors. These networks help in discovering new types of relationships and characteristics. Multiplex networks are realistic representation where they can take all types of information influencing a certain dynamic to predict the outcomes in future.

**Fig. 2:**
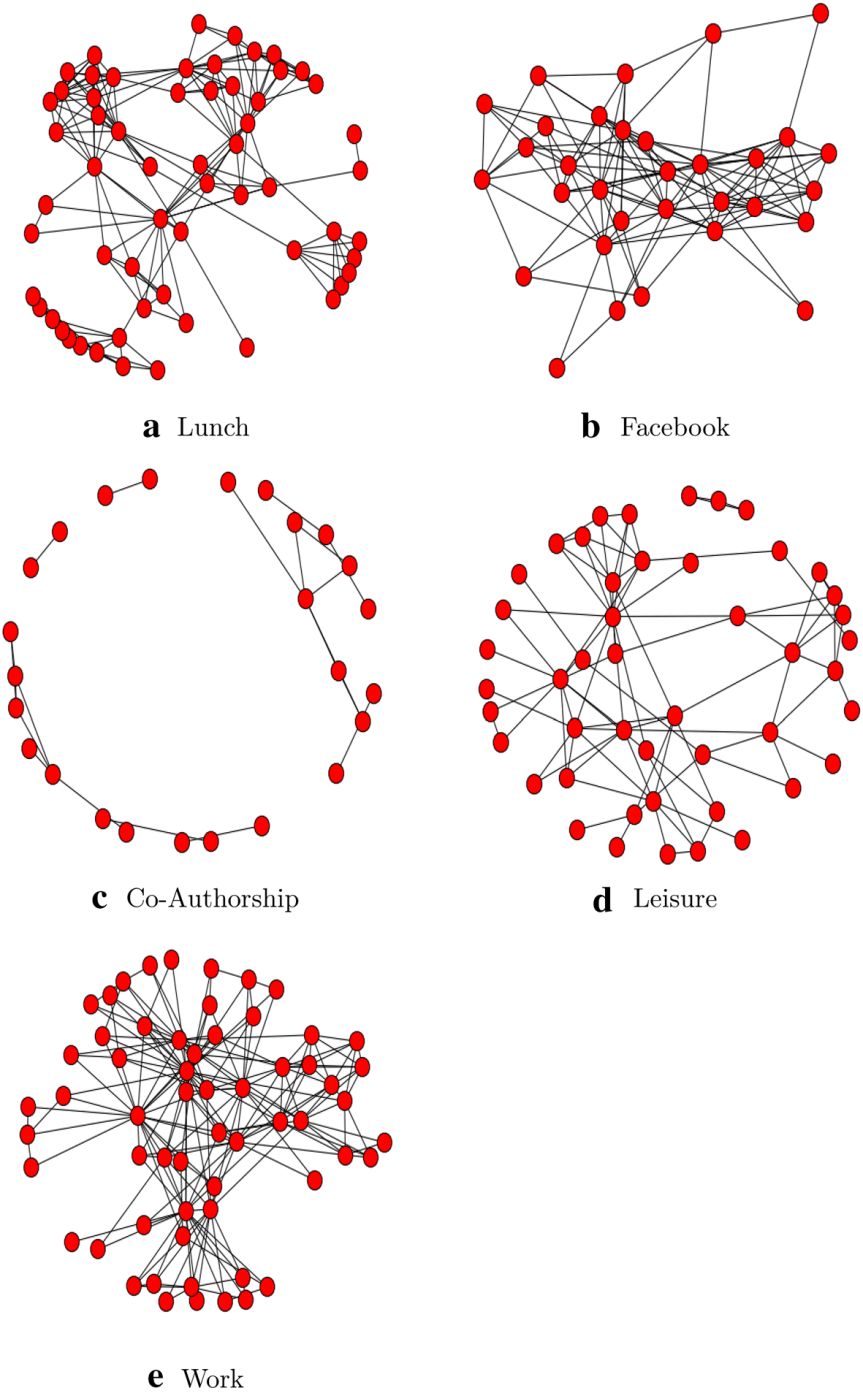
Offline relationships (Facebook, Leisure, Work, Co-authorship, Lunch) between the employees of Computer Science Department at Aarhus

In this work, the multiplex social network consists of five kinds of online and offline relationships (Facebook, Leisure, Work, Co-authorship, Lunch) between the employees of Computer Science department at Aarhus [21] (Fig. 2).

- **Layer 1:** Representative of two individuals having Lunch together.
- **Layer 2:** Representative of two individuals having a social connection via Facebook.
- **Layer 3:** Representative of two individuals co-authoring a publication.
- **Layer 4:** Representative of two individuals having Leisure together.
- **Layer 5:** Representative of two individuals working together.

## Proposed methodology for link prediction in multiplex networks

The proposed method uses the information from all other layers of the network for the purpose of link prediction at an individual layer known as the target layer. Other layers are referred to as predictor layers. Each layer influences prediction on a target layer to a different extent. Some form a good representation of the target layer, whereas others may create a poor representation. This information is required for a good overall link prediction. Relationship and representational information between the target layer and each of the predictor layer are extracted from the network structure and links. This information is tested to get a measure of how well does a particular predictor layer represent the target layer. The final link prediction score is a weighted result of the above outcomes.

More specifically, the final score is assigned as a weighted combination of scores of each layer. The weights are extracted by checking the link correspondence between two layers using likelihood of a link being present in the target layer given the link is present in the predictor layer. This orders the predictor layers concerning relative importance for a specific target layer.

**Fig. 3:**
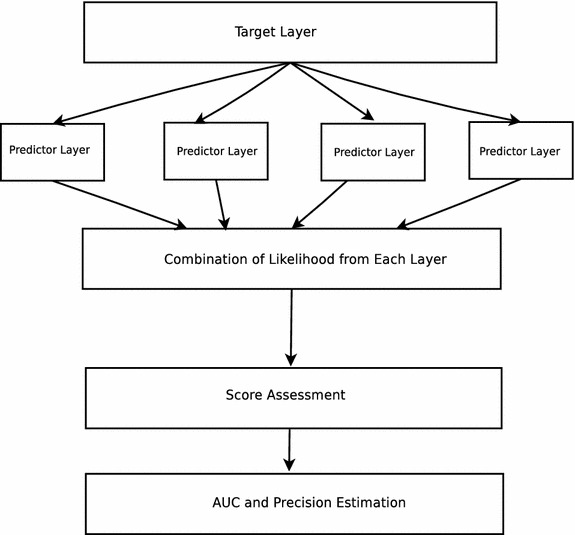
Link prediction for multiplex networks

A pictorial representation of the proposed algorithm is given in Fig. 3. Each predictor layer provides the information about the likelihood of the presence of a link in a target layer. The likelihood is added to assign a score to each pair of nodes. These are then sorted and tested for precision. Similarly, a non-observed link and an observed link are both picked randomly and compared by likelihood score assigned to obtain the AUC.

The first algorithm (Algorithm 1) is used to assign the likelihood of a link existing in a layer based on information obtained from the other layers. For each layer, the likelihood is individually calculated and used as a weight. The overall likelihood is a combination of the likelihoods obtained individually [22]. The likelihood is calculated by observing a previous snapshot (denotes a subset of the graph after removing the edges used for performance testing purposes) of the graph and getting an estimation of the dependency of the probability of the existence of a link in the target layer given a predictor layer. The same is carried out for each pair. The second algorithm (Algorithm 2) is used to find the AUC measure for the proposed method. It picks up two edges in an iterative manner, one from the training set and another edge which was not a part of the graph (non-observed). It compares the likelihoods and gets incremented in case the likelihood of an edge that was predicted correctly is greater than a non-observed edge. The algorithm to obtain precision (Algorithm 3) sorts all edges based on the assigned scores and checks the number of relevant (correctly predicted) edges which contribute to the top of sorted scores.

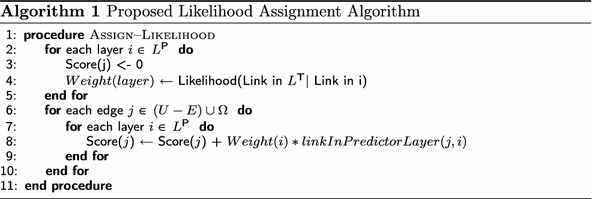



The algorithm inputs a network and returns it by attaching scores to each edge based on a likelihood of formation.

The procedure *linkInPredictorLayer* is used to obtain information if a link is found to be present in a predictor layer at some snapshot of the network. It assesses a predictor layer for the presence of an edge and uses a likelihood estimate to get a prediction for the presence of an edge in the target layer. It returns true (a value of 1) if an edge is present in the predictor layer else it returns a value of 0. The same is repeated for all pairs of layers, and the likelihood is combined to assign a final score. Network structure at an earlier snapshot is known. After obtaining the likelihood and combination of opinion from predictor layers, an idea about the existence of a link in the future can be made.

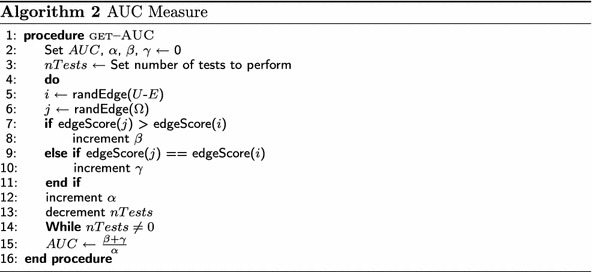

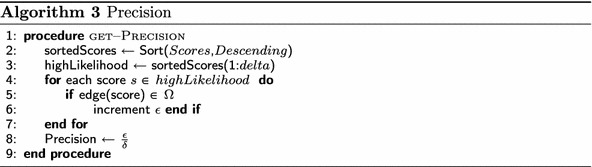





## Results and analysis of proposed link prediction methodology

All of the algorithms mentioned in this work are implemented in Python using the NetworkX module. Tables 2 and 3 list the results of the proposed algorithm.

**Table 2:** Observed AUC values

	CN	JA	PA	Proposed
Layer 1	0.79	0.75	0.71	0.85
Layer 2	0.83	0.84	0.79	0.88
Layer 3	0.1	0.71	0.72	0.8
Layer 4	0.81	0.8	0.79	0.93
Layer 5	0.8	0.82	0.83	0.83

**Table 3:** Observed precision values

	CN	JA	PA	Proposed
Layer 1	0.11	0.43	0.21	0.95
Layer 2	0.33	0.41	0.3	0.83
Layer 3	0.8	0.1	0.2	0.98
Layer 4	0.2	0.11	0.2727	0.61
Layer 5	0.29	0.16	0.29	0.61

In Algorithm 2, randEgde returns a random edge from the augmented Set and edgeScore returns the likelihood of the augmented edge.

Link prediction in networks is of prime importance in various fields. A multiplex network abstracts a variety of heterogeneous information and is closer to reality as decisions are governed by multiple factors rather than just one factor. It is clear from the results that the proposed algorithm outperforms the common neighbor algorithm, preferential attachment, and Jaccard measure in both evaluation measures. In each case, the proposed algorithm has a better AUC value than the other methods. A high AUC is also shown in all layers. The proposed method also has a high and better precision. Even in those cases where the precision for some methods is around 0.27, the proposed method gives a precision of 0.61. On the other hand, in the best case proposed method gives a precision of 0.98 where others have a precision of around 0.8 and 0.43. The tests were also performed on the London multiplex transport network and the proposed algorithm conformed the results obtained on the citation dataset. As the algorithm does not take assumptions (except for the positive correlation between layers) about the network, it is thus context and network independent. Prediction algorithms for single layer network poorly extend on a multiplex network, whereas the proposed algorithm accurately predicts links on it as evidenced by the analysis. Further experimentation is currently in progress. Very few research work has been done in the area of multiplex link prediction for generalized data. An approach based on rank aggregation [23] is proposed for a specific DBLP citation dataset in recent time which assumes certain properties of a social network. However, it is non-dominated about the objective of this work. Still, this work provides a better AUC measure on the citation dataset as reported in the paper.

## Weight prediction

### Weighted networks

Representation of dynamics in the form of networks has multiple applications ranging from social network modeling, traffic flow modeling to rumor dynamics and community detection. A more accurate and realistic representation of various scenarios involves certain weights between the associations. All ties in a network are generally not equal regarding intensity, capacity, and strength. A weight representing the same notion is associated with each edge. It essentially quantifies the degree of association between two specific nodes in case it exists. The usefulness of weighted networks implied the need of weight prediction in networks. Prediction of the development of links coupled with their strengths would provide a better modeling of real-time dynamics. As a simple example, let us assume a flow traffic scenario modeled as a network where nodes represent various places, edges represent if there exists a road connection between them, and weights represent the traffic load. It would be useful to predict which new link could be formed and how it would impact the traffic load.

**Fig. 4:**
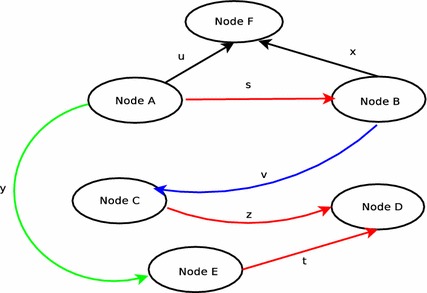
Weighted multiplex example network

Consider, for instance, Fig. 4, which represents the modes of traveling between different nodes/places as described earlier using colored edge representation: road (Red), air (Green), and water (Blue). Assuming each link is bidirectional, let the weights on the links represent the volume of flow from each node. Assuming addition of a new node, *F* is indicated by a link prediction algorithm; it is also important to have an indication of what would be the volume (weight) of the link that will be developed (*u* and *x*); and the type of network that will best suit the cause. For example, if there is a very low volume of commuters to *F*, we could only add just a single road link. This will depend on the weights/volume on other links and their dynamics.

Different methodologies have been used for the purpose of weight prediction in the network. Zhao et al. [24] identify some reliable routes to predict weights. Furthermore, common neighbors, Jaccard and preferential attachment and other methods can also be used for predicting weights utilizing the underlying property they rely on. For example, if two nodes share the high number of common neighbors, the weight of the link between them depends more on the weights of the edge they form with their common neighbors. Similarly, the Jaccard measure assumes higher values for pairs of nodes which share a higher proportion of common neighbors about the total number of neighbors they have biased the weight prediction accordingly. However, these methods take into only account prediction for a single-layered network. We extend the idea from the proposed link prediction algorithm for multiplex networks for the purpose of weight prediction. Weight prediction in the multiplex network is based on the scoring of proposed multiplex likelihood assignment method.

### Dataset

The network used for testing the proposed weight prediction algorithm is the Pierre Auger multiplex network [25]. It consists of layers corresponding to different working tasks within the Pierre Auger collaboration. All submissions between 2010 and 2012 were considered and mapped to various layers according to the keywords and content with manual disambiguation to avoid spurious results from an automated process. The set of nodes represent research work, and there is a link in a particular layer only if they share a common keyword characteristic of that layer. Some keywords are listed in the following table (Table 4). The London multiplex transport network is a three-layer multiplex network where nodes represent train stations in London and edges encode the existing routes between stations: underground, overground, and DLR station. The results are also validated on this network and conform to the ones observed using the Pierre Auger network.

**Table 4:** Description of dataset

Parameter	Description
Number of nodes	514
Number of edges	7153
Number of layers	16
Some keywords	Neutrinos, detector, enhancements, anisotropy, point source

### Proposed methodology for weight prediction in multiplex networks

It is evident from the results that the link prediction algorithm proposed in the work outperforms other indices and is very appropriate for the purpose of link prediction. The scores assigned by the link prediction algorithm are indicative of how similar two nodes are with the particular property. Higher the score of an index between two nodes, more is the likelihood of them forming a link in the future. Hence, the scores are good indicators of the underlying relationship between nodes. A link between two nodes say *x* and *y* has a high likelihood of formation in future according to the proposed link prediction algorithm. The weight of this link would depend on edges which connect the nodes of this link and their neighbors [26]. We find a neighbor of each node which has the highest score. A weighted average is then taken off the weights between the shortlisted neighbors and the corresponding node of the link. The weight is used for normalization in case scores have a substantial difference. It is evident that more layers provide more information (assuming it is non-arbitrary) and hence a better performance.

7 $$\begin{aligned} W_{XY} =\frac{(\text{WT}_{1}*W_{AX}) + ((\text{WT}_{2}*W_{BY})) }{2} \end{aligned}$$

8 $$\begin{aligned} \text{WT}_{1} =(1+((S_{AX}-S_{XY})/S_{AX})) \end{aligned}$$

9 $$\begin{aligned} \text{WT}_{2}= (1+((S_{BY}-S_{XY})/S_{BY})) \end{aligned}$$

where *X* and *Y* are nodes for whose link the weight has to be predicted; *A* and *B* are respective neighbors with highest scores; $W_{XY}$ is the weight of the edge between node X and Y; $W_{AX}$ and $S_{AX}$ denote the weight and score respectively of the edge between node A and X; $\text{WT}_1$ and $\text{WT}_2$ are weights and are used for normalizing in case of large score difference.

**Fig. 5:**
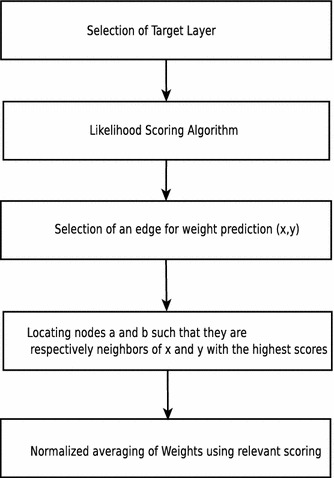
Link prediction and weighting methodology

Combining the accurate link prediction algorithm which uses the multiplex structure and takes into account many factors to predict weights results in accurate weight prediction (Fig. 5).

### Results and analysis for weight prediction

The methodology is tested over a large number of test cases. A parameter of normalized root-mean-square error (NRMSE) was used to quantify the overall error in the actual and predicted weights over the number of test cases. For *n* as the number of test cases, NRMSE it can be represented by the following equation:

10 $$\begin{aligned} = \frac{\sqrt{\frac{1}{n} \sum _{i=1}^{n} (\text{weight}_{\text{predicted}}-\text{weight}_{\text{actual}})^{2} }}{\text{max}(\text{weight}_{\text{predicted}})-\text{max}(\text{weight}_{\text{actual}})} \end{aligned}.$$

Normalized root-mean-square error essentially quantifies the averaged and normalized error between two vectors. In our case, these vectors are the vector of the weight predicted and actual weight for respective edges which were under testing.

The following table shows the NRMSE for different sections of data and layers.

**Table 5:** Observed average NRMSE values

Section	Number of tests	CN	JA	PA	Proposed
1	50	0.0623	0.0801	0.1207	0.0212
2	100	0.084	0.07488	0.10407	0.00955
3	200	0.0455	0.00389	0..0823	0.001919
4	500	0.0076	0.0056	0.0912	0.0017
5	1000	0.01956	0.00790	0.1025	0.00162

The NRMSE is the square root of squared difference of predicted values normalized by number of tests and range of predicted weights [26].

**Fig. 6:**
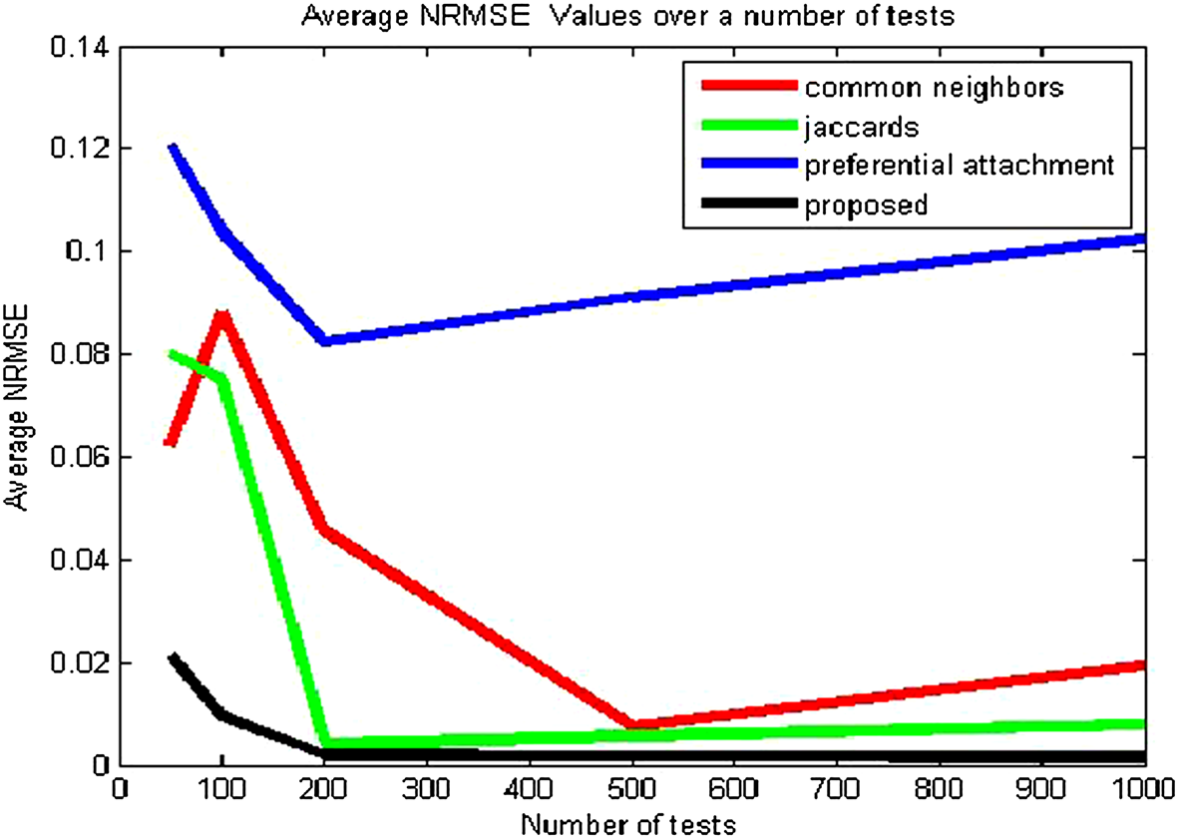
Average NRMSE values for different methodologies

It is evident from Table 5 that the error obtained is relatively small, and the predicted weights are very near to the actual weights; the same is also shown in Fig. 6. The methodology considers effects of other layers by the scoring mechanism. The proposed method performs better regarding a lower NRMSE value as compared to other indices for a different number of test cases. Jaccard and common neighbors produce better results as compared to preferential attachment but still give error values that are more than double than the proposed algorithm in the worst case and around ten to thirty times the proposed algorithm on an average. Over a different number of tests, the proposed method results in better prediction and is scalable in nature. The scoring from multiple layers results in a lesser error. The results are consistent and not sensitive to the parameters of networks. The proposed methodologies are generally applicable over a large range of networks. The trends of NRMSE values over a range of tests are however not significant but exhibit an initial dip and then a slow increase is generally observed. The proposed method results in the lowest error in every instance.

## Conclusions

A network is an abstraction of various kinds of dynamics that govern a process. A model for prediction of these dynamics is of significant importance in many fields viz social networks, biological networks, and transportation networks. Existing link prediction algorithms focuses on a single type of information. A multiplex network facilitates link prediction using information of different types which is a more accurate representation of reality. This work successfully proposes an algorithm for link prediction on generalized multiplex networks. It interprets the relations, and their relative importance in the whole network visualized in the form of multiple layers. The algorithm establishes a likelihood of the presence of edges in one layer with respect to the other layer. This process is repeated for all layers to obtain a likelihood measure individually. The combination of the likelihoods is taken to pass final judgment. The method uses multilayer information rather than the intralayer information. The validation proves that the algorithm outperforms the common neighbor, Jaccard’s, preferential attachment and provides validation on some intuitive observations. Multiplex networks are closer to reality as they encapsulate various heterogeneous relations rather than just one. The algorithm successfully employs information from all of the layers and combines to give favorable link prediction results on a multiplex network. Furthermore, combined with the target layer information, the algorithm can prove to be an extension of single layer indices over multiplex networks. The proposed likelihood assignment algorithm for link prediction performs well over a multiplex dataset. The network assumes the positive correlation between relations depicted by each layer. It is used for scoring a network to obtain similar edges whose weights are further averaged in a normalized manner to assign a weight to an edge. The predicted weights have very less deviation from actual weights. Compared to other indices, the proposed method has a far low error rate and outperforms them concerning the metric performance NRMSE.
